# The Opioid-Sparing Benefits of Regional Anesthesia With Sedation in Pediatric Orthopedic Surgery: A Retrospective Analysis

**DOI:** 10.7759/cureus.101489

**Published:** 2026-01-13

**Authors:** Raphael Israeli, Sarina Sheiner, Yuliya Gadulov, Amir Herman, Lilach Hazan, Omri Barkan

**Affiliations:** 1 Department of Orthopedic Surgery, Kaplan Medical Center, Rehovot, ISR; 2 Department of Anesthesiology, Kaplan Medical Center, Rehovot, ISR; 3 Department of Anesthesiology, Shamir Medical Center, Be'er Ya'akov, ISR; 4 Department of Plastic and Reconstructive Surgery, Kaplan Medical Center, Rehovot, ISR

**Keywords:** anesthesia, opioid analgesics, orthopedic surgery, pediatric, postoperative pain, regional

## Abstract

Introduction

Regional anesthesia may reduce perioperative opioid exposure in pediatric orthopedic surgery; however, comparative data with general or combined anesthesia remain limited. This study evaluated the association between regional anesthesia with sedation (RA) and perioperative opioid consumption, as well as its relationship with early recovery outcomes, compared to general or combined anesthesia.

Methods

We performed a retrospective cohort study of 191 pediatric patients (0-18 years) who underwent single-limb orthopedic surgery between 2019 and 2022 at Kaplan Medical Center. Patients were stratified by anesthetic technique: general anesthesia (GA, n = 130), combined general and regional anesthesia (GA+RA, n = 32), and RA (n = 29). The primary outcome was intraoperative opioid consumption, expressed as morphine equivalent dose (MED) per kg. Secondary outcomes included postoperative opioid use in the post-anesthesia care unit (PACU) and ward, Face, Legs, Activity, Cry, Consolability (FLACC) pain scores, postoperative nausea and vomiting (PONV), PACU length of stay, and total hospital stay.

Results

Median intraoperative opioid use was significantly lower in the RA group (0.16, IQR 0.11-0.20) than in the GA+RA (0.26, IQR 0.18-0.32) and GA groups (0.30, IQR 0.16-0.38) (p < 0.001). PACU opioid consumption was also reduced in the RA and GA+RA groups (0.01 MED/kg each) compared to the GA group (0.03 MED/kg; p = 0.001). A FLACC score of 0 in the PACU was more frequent in the RA group (72.4%) than in the GA+RA (62.5%) and GA (47.7%) groups (p = 0.042). The incidence of PONV was lowest in the RA group (3.4%), although the difference was not statistically significant (p = 0.365).

Conclusions

RA was associated with significantly lower intraoperative and immediate postoperative opioid requirements and improved early pain control compared with GA. These findings support the broader adoption of sedation-assisted regional anesthesia as an opioid-sparing strategy in pediatric orthopedic surgery.

## Introduction

General anesthesia (GA) has traditionally been the primary anesthetic technique used in pediatric patients [1]. Over the past decade, ultrasound-guided peripheral nerve blocks (PNBs) have gained traction, with growing evidence supporting their role in improving postoperative outcomes by reducing opioid consumption and enhancing analgesia [2,3]. Current guidelines recommend performing regional blocks under GA or deep sedation to reduce discomfort and minimize patient movement [2].

Despite these advancements, most pediatric surgeries continue to be conducted under GA alone, with only a small proportion using a combination of general and regional anesthesia techniques. Even fewer procedures are performed under regional anesthesia as the primary anesthetic, often in combination with sedation rather than GA [4]. This limited adoption likely reflects institutional preferences and the current lack of large-scale comparative evidence to support the routine use of regional anesthesia with sedation (RA) in pediatric surgery.

While the safety profile of pediatric regional anesthesia is well established [3], comprehensive comparative data quantifying its specific opioid-sparing efficacy versus GA in pediatric orthopedic surgery remain limited [5,6]. Although regional anesthesia has been studied in specific settings, such as neonatal hernia repair, where it was associated with reduced postoperative apnea and opioid exposure [7], there remains a need for high-quality data comparing RA to other standard techniques in more painful procedures, such as pediatric orthopedic surgery. Establishing this comparison is clinically important to determine whether the systemic risks of GA, specifically airway manipulation and emergence delirium, can be effectively avoided without compromising analgesic quality.

Orthopedic procedures, particularly those involving fractures or soft-tissue repair, are associated with considerable postoperative pain, requiring effective multimodal analgesia [8,9]. Current research indicates that regional anesthesia provides superior analgesia while minimizing opioid-associated adverse events compared with local infiltration [10]. However, no studies have specifically examined whether pediatric patients undergoing orthopedic surgery with regional anesthesia under sedation require lower postoperative opioid doses than those receiving general or combined anesthesia. Determining the comparative efficacy of these techniques is clinically essential. While perioperative opioid dosages in pediatric patients are typically lower than in adults, children exhibit heightened sensitivity to opioid-related adverse events. Therefore, minimizing opioid exposure is a central tenet of modern pediatric Enhanced Recovery After Surgery (ERAS) protocols [11], aiming not only to reduce the risk of potential dependence but also to mitigate immediate complications, such as respiratory depression and postoperative nausea and vomiting (PONV). These complications are significant drivers of patient distress and prolonged hospital length of stay, necessitating anesthetic strategies that maintain analgesic quality while minimizing systemic opioid burden [12].

To address this gap, the present retrospective study investigated the association between anesthetic technique and perioperative opioid consumption in pediatric orthopedic surgery. The primary objective was to determine whether children who underwent RA required lower intraoperative opioid doses than those who received GA alone or combined general and regional anesthesia (GA+RA). We hypothesized that RA would be associated with reduced opioid use, improved pain control, and fewer opioid-related side effects than GA. Secondary outcomes included postoperative opioid use, pain scores, PONV, recovery duration, and hospital stay, further defining the clinical impact of each anesthetic approach on pediatric recovery and opioid stewardship.

## Materials and methods

Study design

This retrospective cohort study was conducted at Kaplan Medical Center and analyzed pediatric orthopedic patients who underwent surgery between June 1, 2019, and March 1, 2022. Ethical approval was obtained from the Kaplan Medical Center Institutional Helsinki Committee (approval no. KMC-0103-25). The study was conducted in accordance with the Declaration of Helsinki (2013 revision).

Study population

Eligible participants included pediatric patients from infancy to 18 years of age who underwent orthopedic procedures such as fracture fixation, hardware removal, or tendon transfer involving a single limb. Exclusion criteria were minor procedures (e.g., trigger finger release, Achilles tenotomy, and single screw removal), polytrauma, multiple simultaneous surgeries, incomplete documentation of anesthesia technique or postoperative analgesia, and absence of informed consent from legal guardians.

Data collection

Demographic and clinical data were extracted retrospectively from electronic medical records and operating room logs. Collected variables included age, weight, sex, surgical procedure, attending surgeon and anesthesiologist, type of anesthesia, airway management, and use of PNBs. Opioid consumption was recorded intraoperatively, in the post-anesthesia care unit (PACU), and on the ward, expressed as morphine equivalent dose (MED) per kilogram of body weight. Additional outcomes included postoperative pain scores using the Face, Legs, Activity, Cry, Consolability (FLACC) scale, incidence of PONV, PACU length of stay, and total hospital length of stay. All data were anonymized before analysis.

The FLACC pain assessment tool was used under a valid academic and research use license (license no. 71EAT) issued by the University of Michigan Office of Technology Transfer (file 6581). Although traditionally validated for younger children, the FLACC scale was utilized in this study to assess pain during the immediate emergence phase in the PACU, where verbal self-reporting is often unreliable due to residual sedation regardless of patient age.

Anesthesia groups

Patients were categorized into three anesthesia groups based on the type of anesthesia administered. Allocation to the anesthesia groups was non-randomized and determined by the attending anesthesiologist’s preference and surgical complexity. The RA group received ultrasound-guided PNBs tailored to the surgical site. For upper extremity procedures, infraclavicular or interscalene blocks were utilized. For lower extremity procedures, popliteal (sciatic), femoral, or adductor canal blocks were administered. The standard local anesthetic solution used was 0.25% bupivacaine (Marcaine). In selected cases requiring faster onset, a mixture of bupivacaine and lidocaine was administered. No adrenaline was added to the solution. To ensure safety and prevent local anesthetic systemic toxicity, the total dosage was strictly calculated based on patient weight, with a maximum limit of 2 mg/kg of bupivacaine. Sedation in the RA group was maintained using propofol exclusively, administered as an initial bolus followed by intermittent boluses or continuous infusion to maintain deep sedation while preserving spontaneous ventilation. The use of benzodiazepines, ketamine, or other sedative adjuncts was a strict exclusion criterion for the RA group in this analysis to ensure protocol uniformity. The GA group included patients who were administered GA (using a laryngeal mask airway or endotracheal tube) without the addition of regional techniques. Finally, the combined anesthesia (GA+RA) group consisted of patients who received both GA and regional anesthesia, typically with the nerve block administered after induction.

Intraoperative analgesia across all groups was maintained primarily using intravenous fentanyl and morphine. Opioid administration was titrated to clinical effect based on standard hemodynamic monitoring of nociception (e.g., tachycardia >20% above baseline or hypertension). To ensure standardized comparisons, all opioid dosages were converted to and recorded as MED per kilogram of body weight. Calculations were based on standard equianalgesic conversion factors: intravenous fentanyl 10 mcg was considered equivalent to 1 mg of intravenous morphine.

Outcomes

The primary outcome was intraoperative opioid consumption, expressed as MED/kg. Secondary outcomes included opioid use in the PACU and ward, FLACC pain scores, incidence of PONV, PACU length of stay, and total hospital stay.

Statistical analysis

Continuous variables were tested for normality using the Shapiro-Wilk test. As a normal distribution could not be assumed, non-parametric statistical tests were applied. Comparisons among the three anesthesia groups were performed using Brown-Forsythe and Welch’s ANOVA tests for continuous variables, with Games-Howell post hoc testing for pairwise comparisons. Categorical variables were expressed as frequencies and percentages and analyzed using Pearson’s chi-square or Fisher’s exact test, as appropriate. Missing data were minimal and excluded on a case-wise basis. Statistical significance was defined as p < 0.05. Analyses were performed using IBM SPSS Statistics for Windows, Version 22.0 (Released 2013; IBM Corp., Armonk, NY, USA).

## Results

A total of 223 medical records of pediatric orthopedic surgeries performed between June 1, 2019, and March 1, 2022, were screened. After applying the exclusion criteria (minor procedures, polytrauma, or incomplete documentation), 32 patients were excluded. The final analysis included 191 patients (GA, n = 130; GA+RA, n = 32; RA, n = 29). Baseline demographic and clinical characteristics of the anesthesia groups are summarized in Table 1.

**Table 1:** Pediatric patient demographics and clinical characteristics by type of anesthesia Data are presented as median (25th-75th percentile) or n (%). Statistical significance is indicated as follows:^ **^ p < 0.01. A hyphen (-) denotes no applicable data. Continuous variables were analyzed using the Kruskal-Wallis H test (H statistic reported). Categorical variables were analyzed using Pearson’s chi-square (χ²) test. Statistical significance was set at p < 0.05. ASA, American Society of Anesthesiologists; CR, closed reduction; CRIF, closed reduction internal fixation; GA, general anesthesia; GA+RA, combined general and regional anesthesia; ORIF, open reduction internal fixation; RA, regional anesthesia with sedation

Characteristic	GA	GA+RA	RA	Test statistic	p-Value
Demographics					
Age (median (IQR)), years	9.04 (6.25-12.7)	10.61 (7.95-13.1)	10.88 (7.24-13.1)	H(2) = 1.69	0.430
Sex, n (%)				χ²(2) = 1.73	0.422
Female	48 (36.9%)	8 (25%)	11 (38%)	-	-
Male	82 (63.1%)	24 (75%)	18 (62%)	-	-
ASA classification, n (%)				χ²(4) = 1.60	0.806
ASA I	86 (82.7%)	21 (84.0%)	18 (75.0%)	-	-
ASA II	16 (15.4%)	4 (16.0%)	5 (20.8%)	-	-
ASA III	2 (1.9%)	0 (0.0%)	1 (4.2%)	-	-
Clinical characteristics					
Surgery type, n (%)				χ²(4) = 17.99	<0.001^**^
CRIF/CR	76 (58.5%)	7 (21.9%)	11 (37.9%)	-	-
ORIF	25 (19.2%)	13 (40.6%)	12 (41.4%)	-	-
Other	29 (22.3%)	12 (37.5%)	6 (20.7%)	-	-

Primary outcome

**Figure 1:**
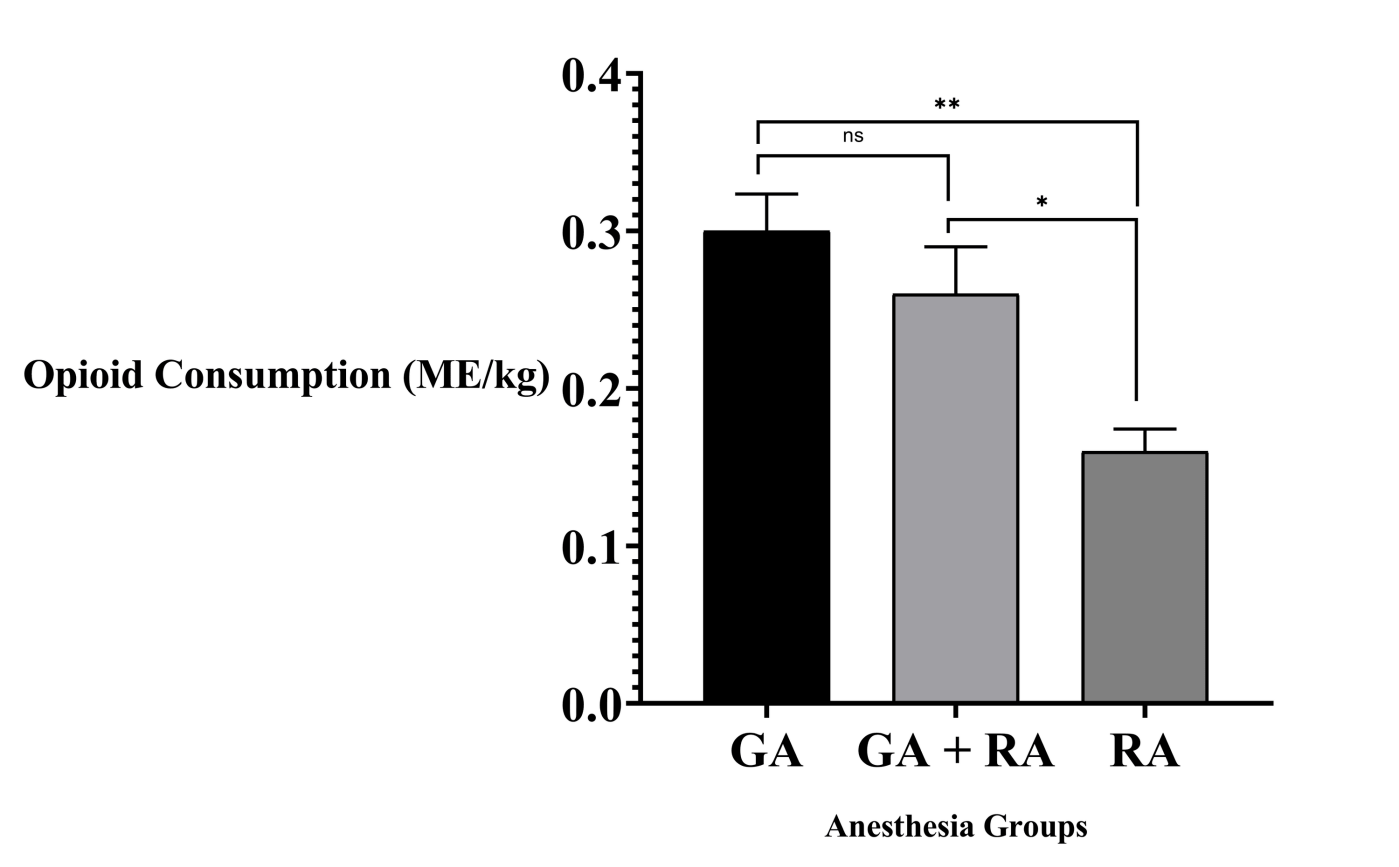
Intraoperative opioid consumption by anesthesia type (MED/kg) Statistical significance is indicated as follows: ^*^ p < 0.05, ^**^ p < 0.01, ns = not significant GA, general anesthesia; GA+RA, combined general and regional anesthesia; MED, morphine-equivalent dose; RA, regional anesthesia with sedation

Intraoperative opioid consumption, measured as MED per kilogram, differed significantly among the anesthesia groups (H(2) = 12.889, p = 0.002). Median intraoperative opioid use was lowest in the RA group (0.16 (0.11-0.20) mg/kg), followed by GA+RA (0.26 (0.18-0.32) mg/kg), and highest in the GA group (0.30 (0.16-0.38) mg/kg). These results are illustrated in Figure 1 and detailed in Table 2.

**Table 2:** Perioperative outcomes by type of anesthesia Data are presented as median (25th-75th percentile) or n (%). Statistical significance is indicated as follows: ^*^ p < 0.05, ^** ^p < 0.01, ^***^ p < 0.001. A hyphen (-) denotes no applicable data. Continuous variables were analyzed using the Kruskal-Wallis H test (H-statistic reported). Categorical variables were analyzed using Pearson’s chi-square (χ²) test. Statistical significance was set at p < 0.05. FLACC, Face, Legs, Activity, Cry, Consolability; GA, general anesthesia; GA+RA, combined general and regional anesthesia; ME, morphine equivalent; PACU, post-anesthesia care unit; PONV, postoperative nausea and vomiting; RA, regional anesthesia with sedation

Outcome	GA	GA+RA	RA	Test statistic	p-Value
Opioid consumption, median (IQR) (MED/kg)					
Intraoperative	0.30 (0.16-0.38)	0.26 (0.18-0.32)	0.16 (0.11-0.20)	H(2) = 12.889	0.002^**^
PACU	0.03 (0.00-0.05)	0.01 (0.00-0.00)	0.01 (0.00-0.00)	H(2) = 11.329	0.003^**^
Ward	0.07 (0.00-0.00)	0.04 (0.00-0.00)	0.06 (0.00-0.00)	H(2) = 0.498	0.78
Pain scores					
Max FLACC in PACU, n (%)				χ²(4) = 20.36	<0.001^***^
FLACC = 0	62 (47.7%)	20 (63%)	21 (72%)	-	-
FLACC 1-4	36 (27.7%)	7 (22%)	5 (17%)	-	-
FLACC ≥5	32 (24.6%)	5 (16%)	3 (10%)	-	-
Complications					
PONV, n (%)	14 (11.0%)	2 (6.3%)	1 (3.4%)	χ²(2) = 2.01	0.365
Recovery outcomes					
Hospital length of stay (days), median (IQR)	2.00 (2.00-3.00)	3.00 (2.00-4.00)	3.00 (2.00-4.25)	H(2) = 7.02	0.03^*^

Secondary outcomes

**Figure 2:**
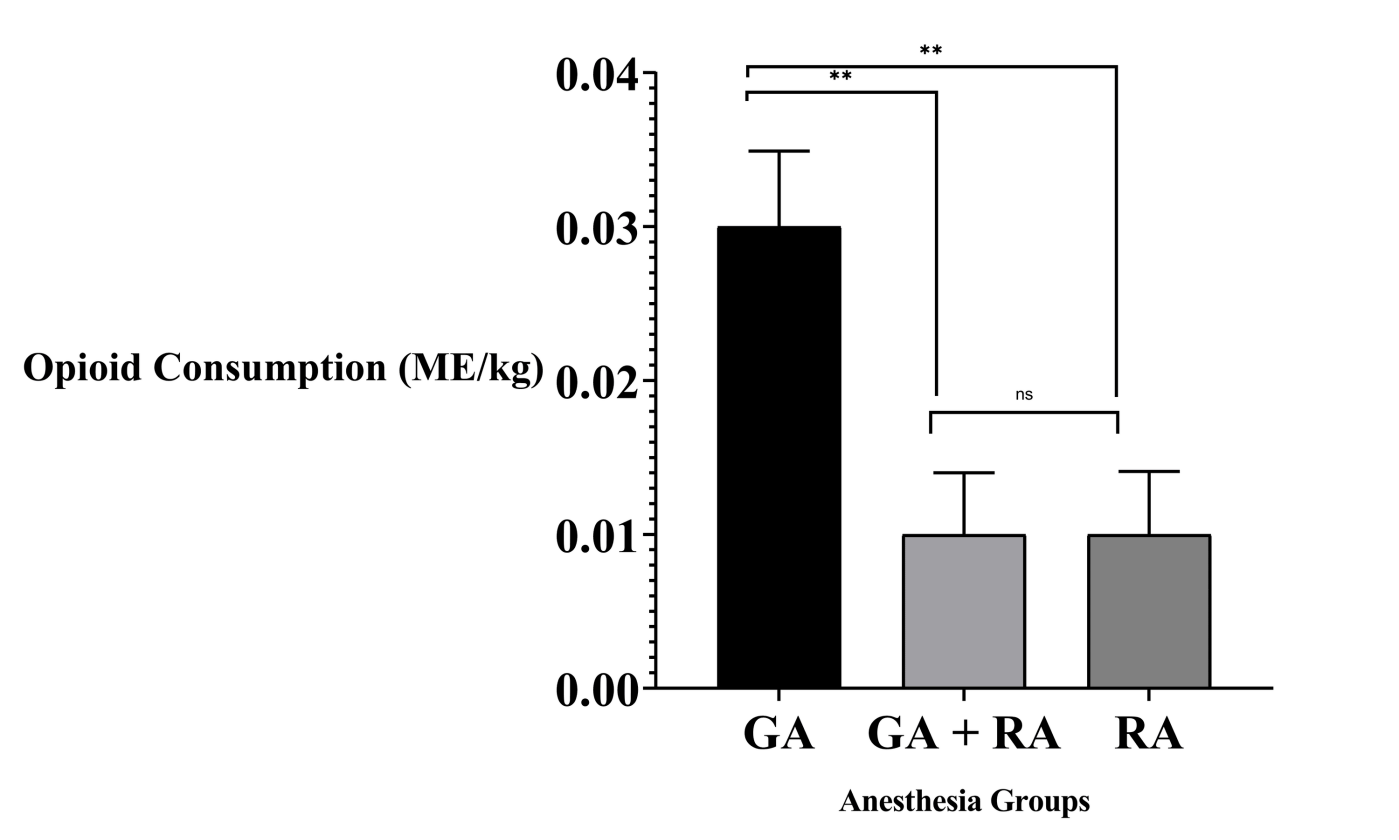
PACU opioid consumption by anesthesia type (MED/kg) Significance markers: ^**^ p < 0.01; ns = not significant GA, general anesthesia; GA+RA, combined general and regional anesthesia; MED, morphine equivalent dose; PACU, post-anesthesia care unit; RA, regional anesthesia with sedation

Postoperative opioid use was assessed in both the PACU and the inpatient surgical ward. In the PACU, opioid consumption was significantly lower in the RA and GA+RA groups compared with the GA group (H(2) = 11.329, p = 0.003). Median PACU opioid consumption was 0.01 (0.00-0.00) mg/kg in the RA and GA+RA groups versus 0.03 (0.00-0.05) mg/kg in the GA group (Figure 2).

In contrast, opioid consumption on the surgical ward did not differ significantly among the groups (H(2) = 0.498, p = 0.78), with all groups showing minimal or no opioid use (median 0.00 (0.00-0.00) mg/kg). Detailed perioperative opioid data are presented in Table 2.

Pain intensity in the PACU, assessed using the FLACC scale, differed significantly among anesthesia groups (χ²(4) = 20.36, p < 0.001). Patients in the RA group predominantly exhibited FLACC scores of 0-3, indicating mild or no pain, whereas higher pain scores (4-10) were more frequent in the GA group. The distribution of FLACC categories is presented in Table 2.

The incidence of PONV was lowest in the RA group (3.4%), followed by GA+RA (6.3%) and GA (11.0%). Although the RA group showed a favorable trend, the difference was not statistically significant (χ²(2) = 2.01, p = 0.365).

Recovery outcomes showed mixed results. PACU length of stay did not differ significantly among the groups; however, total hospital length of stay varied significantly (H(2) = 7.02, p = 0.03). Median hospital stay was shorter in the GA group (2.0 days (2.0-3.0)) compared with the RA group (3.0 days (2.0-4.3)) and the GA+RA group (3.0 days (2.0-4.0)). Full recovery data are summarized in Table 2.

## Discussion

Main findings 

In this retrospective cohort study of pediatric patients undergoing single-limb orthopedic surgery, RA was associated with significantly lower intraoperative opioid consumption compared with both GA and GA+RA. This opioid-sparing effect extended into the immediate postoperative period, with patients in the RA and GA+RA groups requiring less opioid analgesia in the PACU. Superior early postoperative pain control was also observed in the RA group, with a higher proportion of patients achieving a PACU FLACC score of 0.

Comparison with previous literature 

These findings reinforce and expand upon existing evidence supporting regional anesthesia as a cornerstone of opioid-sparing strategies in pediatric surgery [3,13-16]. While prior studies have demonstrated the benefits of regional techniques in various contexts, such as neonatal hernia repair, our study provides specific comparative data in pediatric orthopedic surgery, which is associated with a high burden of postoperative pain [7]. Our results are consistent with those of Xie et al. (2024), who reported significant opioid reduction with regional anesthesia in a large pediatric cohort [3], and DelPizzo et al. (2020), who found that primary regional anesthesia yielded the most substantial opioid reduction [4]. Collectively, these findings confirm that regional techniques provide effective analgesia even for procedures with substantial nociceptive burden [17,18].

Clinical implications 

Beyond reducing opioid use, RA was associated with improved early recovery and lower pain scores compared with GA and GA+RA, consistent with prior studies demonstrating superior analgesia with regional techniques [2,19,20]. The incidence of PONV showed a favorable trend in the RA group, although this difference did not reach statistical significance. Conversely, total hospital length of stay was significantly longer in the RA and GA+RA groups. This likely reflects selection bias, as the RA group included a higher proportion of complex open surgeries (open reduction internal fixation, ORIF), whereas the GA group primarily comprised closed reductions. These findings align with pediatric ERAS principles, which emphasize minimizing opioid exposure, preventing PONV, and promoting faster recovery [10,21,22].

Strengths and limitations 

These results should be interpreted in the context of the study’s strengths and limitations. A key strength of this study is the inclusion of three distinct anesthesia groups, allowing direct comparison of standalone regional anesthesia with general and combined anesthesia techniques. The use of strict weight-based normalization for opioid consumption (MED/kg) and local anesthetic dosage enabled standardized comparisons across a physiologically diverse pediatric population [23,24]. Additionally, the inclusion of a wide range of orthopedic procedures enhances the generalizability of the findings to various surgical contexts.

However, several limitations must be acknowledged. First, the retrospective design inherently limits control over confounding variables. Allocation to anesthesia groups was not randomized but determined by provider preference and clinical judgment, introducing selection bias. Multivariable regression analysis to adjust for potential confounders, such as age or surgical duration, was not performed due to the limited sample size in the RA group (n = 29), precluding robust statistical modeling. Surgical distribution differed significantly between groups (p < 0.001), with more invasive procedures (e.g., ORIF) occurring more frequently in the RA and GA+RA groups. Notably, despite this bias toward more complex surgeries, the RA group demonstrated superior opioid-sparing effects.

Second, although the MED is a standard metric for comparative opioid research, it accounts for analgesic potency rather than pharmacokinetics or duration of action. Because both fentanyl (short-acting) and morphine (long-acting) were used, MED calculations may not fully capture the temporal profile of analgesia.

Third, the FLACC scale was applied across the entire cohort to maintain consistency during the immediate PACU emergence phase. While the FLACC scale is validated primarily for children aged two months to seven years, its use in older children represents a limitation; however, this was necessary due to the unreliability of verbal self-reporting during recovery from deep sedation.

Finally, reliance on electronic medical records introduces the possibility of incomplete documentation. Future studies should adopt a prospective, randomized design to address these limitations.

Future directions

Future prospective trials should aim to confirm these findings and assess long-term outcomes, including chronic post-surgical pain and cost-effectiveness [25,26]. The emerging field of pharmacogenetics may further enable personalized anesthesia protocols, optimizing both safety and analgesic efficacy in pediatric populations [27,28].

## Conclusions

Our findings suggest that regional anesthesia with propofol sedation is an effective anesthetic strategy for pediatric orthopedic surgery. This approach aligns with ERAS protocols, as it is associated with significantly lower perioperative opioid requirements and a trend toward fewer immediate adverse effects. While long-term opioid dependence remains a broader public health concern, the immediate clinical benefits of opioid-sparing techniques, specifically improved recovery quality and favorable safety profiles, support broader adoption. Achieving these benefits requires institutional support, access to ultrasound-guided equipment, and structured training for providers to ensure both safety and efficacy.
